# Forced Mineral Carbonation of MgO Nanoparticles Synthesized by Aerosol Methods at Room Temperature

**DOI:** 10.3390/nano13020281

**Published:** 2023-01-09

**Authors:** Kyungil Cho, Yeryeong Kang, Sukbyung Chae, Changhyuk Kim

**Affiliations:** 1 School of Civil and Environmental Engineering, Pusan National University, Busan 46241, Republic of Korea; 2 Department of Mechanical Engineering, Korea University of Technology and Education, Cheonan 31253, Republic of Korea

**Keywords:** MgO, nanoparticles, mineral carbonation, external force, hydrated magnesium carbonates

## Abstract

Magnesium oxide (MgO) has been investigated as a wet mineral carbonation adsorbent due to its relatively low adsorption and regeneration temperatures. The carbon dioxide (CO_2_) capture efficiency can be enhanced by applying external force on the MgO slurry during wet carbonation. In this study, two aerosol-processed MgO nanoparticles were tested with a commercial MgO one to investigate the external force effect on the wet carbonation performance at room temperature. The MgO nano-adsorbents were carbonated and sampled every 2 h up to 12 h through forced and non-forced wet carbonations. Hydrated magnesium carbonates (nesquehonite, artinite and hydromagnesite) were formed with magnesite through both wet carbonations. The analyzed results for the time-dependent chemical compositions and physical shapes of the carbonation products consistently showed the enhancement of wet carbonation by the external force, which was at least 4 h faster than the non-forced carbonation. In addition, the CO_2_ adsorption was enhanced by the forced carbonation, resulting in a higher amount of CO_2_ being adsorbed by MgO nanoparticles than the non-forced carbonation, unless the carbonation processes were completed. The adsorbed amount of CO_2_ was between the maximum theoretical amounts of CO_2_ adsorbed by nesquehonite and hydromagnesite.

## 1. Introduction

Climate change caused by global warming is currently a severe global environmental issue, which brings out extreme weather events such as heat waves and large storms and threatens human lives on the earth [1]. Greenhouse gases in the atmosphere have been known to accelerate global warming and climate change. Carbon dioxide (CO_2_) produced by fossil fuel combustion is a well-known greenhouse gas with the largest share of radiative forcing since 1990 [2]. Therefore, many countries have struggled to achieve carbon neutral status for a sustainable environment and survival. Replacing the fossil fuel with hydrogen (H_2_) is a promising way to accomplish a carbon neutral status. Ideally, H_2_ should be synthesized using renewable energy (green H_2_) [3]. However, most H_2_ is currently produced by the reforming of methane (gray H_2_) with a huge amount of CO_2_ (10 kg CO_2_/1 kg H_2_) [4]. This requires carbon capture and utilization/storage (CCUS) technology to prohibit CO_2_ emission to the atmosphere after the gas reforming (blue H_2_) [5,6]. Mineral carbonation using alkaline earth metal oxides, such as magnesium and calcium oxides (MgO and CaO), is a promising CCUS technology for achieving carbon neutrality and net zero emissions [7]. In particular, MgO has been spotlighted and investigated as a CO_2_ adsorbent because it requires less energy consumption for regeneration than calcium oxide (CaO) [8]. It can also enhance the lifetime of the CO_2_ adsorbent by reducing the thermal fatigues during the regeneration. In addition, controlling indoor CO_2_ concentration is important for human health because people spend more than 80% of a day in an indoor environment [9]. In particular, multi-purpose facilities including kindergartens and schools are required to limit the indoor CO_2_ concentration for the sake of maintaining children’s health, as they cannot easily figure out if the quality of the air is bad and ventilate the indoor air effectively. However, no instruments are currently available that can reduce the indoor CO_2_ concentration below the indoor air quality standard. Therefore, carbon capture technologies should also be developed to improve the indoor air quality. Mineral carbonation at room temperature can be a solution to control the CO_2_ concentration for sustainable indoor air quality.

Many researchers have investigated basic metal oxides (e.g., CaO and Li_2_O) as CO_2_ absorbents at >500 °C [10,11]. MgO-based materials were investigated in a temperature range of 200~350 °C [10,12]. These temperature ranges were related to CO_2_ post-capture in fossil fuel power plants. If the CO_2_ adsorption of adsorbents can be facilitated even at room temperature, the materials can be applied to an indoor air environment where high CO_2_ concentration (>1000 ppm) may cause health problems.

Doping MgO-based adsorbents with transition and alkaline metals is one of the promising approaches to enhance the performance of CO_2_ capture [10,13]. Decreasing particle size and forming porous structures to obtain higher surface areas are also effective methods to enhance CO_2_ absorption since they lead to there being more reaction sites in MgO particles [14,15,16,17,18,19]. Nanostructured MgO/Al_2_O_3_ aerogel [20] and synthesizing MgO via hydrothermal methods with urea [21] optimized the basicity of particle surfaces to increasing CO_2_ adsorption. Vacancy defects in an MgO crystal’s structure have the possibility of increasing CO_2_ uptake by lowering the bonding energy of CO_2_ and the surfaces of MgO crystals [22]. The sizes and molecular structures of MgO nanoparticles can be controlled by the synthesis methods and conditions. Aerosol synthesis methods such as spray pyrolysis, self-combustion and flame metal combustion have high productivity and size controllability, releasing less pollution than that of wet-based chemistry [23]. In addition, cubic or terrace-shaped MgO nanoparticles can be generated via two different combustion methods, such as self-combustion and flame metal combustion methods, respectively [24,25]. External forces such as the mechanical agitation of solutions can also promote the CO_2_ adsorption reaction [26]. By applying the external forces on the mineral carbonation of the MgO nanoparticles, we can optimize and enhance CO_2_ adsorption in a lower temperature range with the same materials.

In this study, three MgO nanoparticles (two kinds of nanoparticles synthesized by two aerosol methods and one commercial nanoparticle) were investigated to figure out the effects of the forced wet carbonation on the carbonation performance of the MgO nanoparticle adsorbents at room temperature. Pairs of each MgO adsorbent prepared as slurry were exposed to a CO_2_ environment in a closed chamber with and without stirring to compare the forced and non-forced wet carbonations.

## 2. Materials and Methods

### 2.1. Preparation and Wet Carbonation Experiments of MgO Nanoparticles

Three kinds of MgO nanoparticles (A-, C- and T-MgO) were prepared by different aerosol methods as adsorbents for wet mineral carbonation. A-MgO represents commercial MgO nanoparticles (<50 nm, Sigma Aldrich Co., Ltd, Saint Louis, MO, USA), and it might be synthesized by an aerosol method based on the morphology. C-MgO means cubic-shaped ones synthesized by the self-combustion method with magnesium chips (99/98% purity, 6–35 mesh, Sigma Aldrich Co., Ltd., Saint Louis, MO, USA) [8,27]. T-MgO is a terrace-shaped nanoparticle, which was produced by the flame metal combustion (FMC) method using a hydrogen/oxygen diffusion flame burner and micro-sized magnesium powders (<45 μm, 98.5% purity, Daejung Chemical and Metals Co., Ltd, Sihueng, Republic of Korea) [23,24,25].

**Scheme 1:**
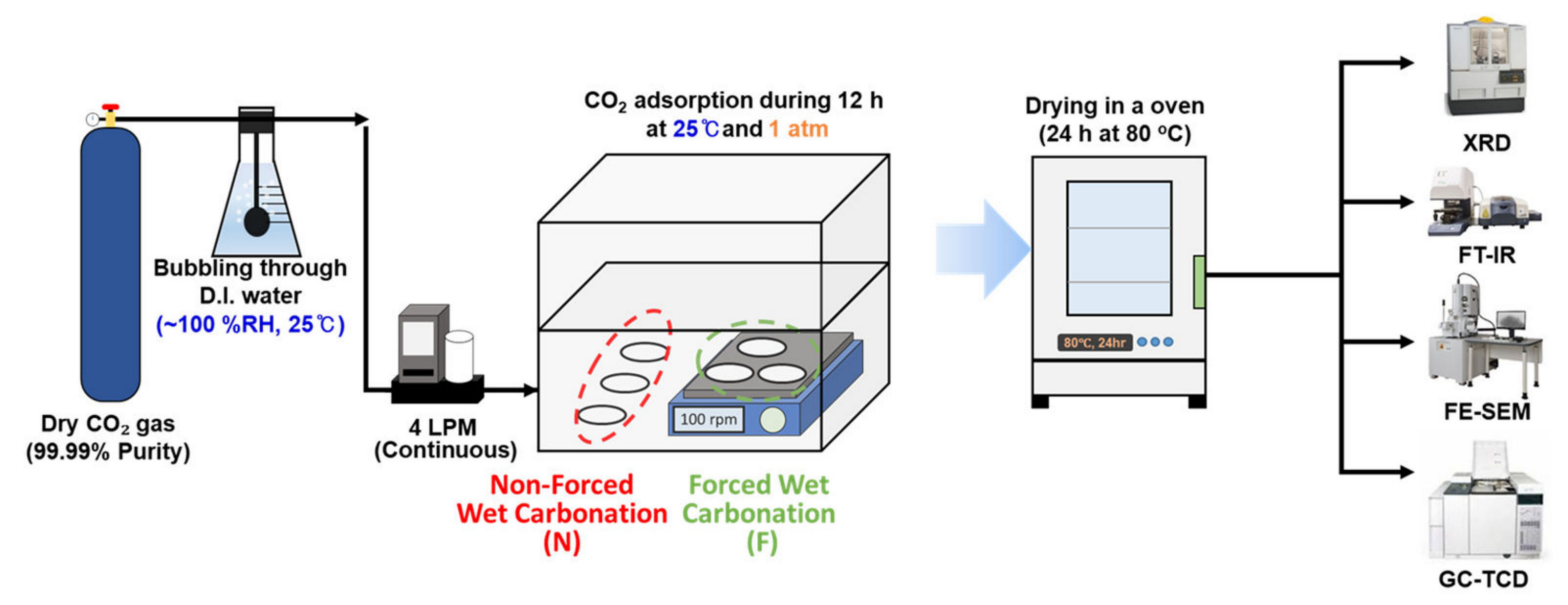
Experimental setup for the forced or non-forced wet carbonations of 3 MgO nanoparticle adsorbents.

Scheme 1 shows the experimental setup for the wet carbonation of the 3 MgO nanoparticle-based adsorbents. The nano-adsorbents were prepared as slurries by mixing 500 mg MgO nanoparticles with 5 mL D.I. water in a glass petri dish. The 3 MgO nano-adsorbents were simultaneously exposed to CO_2_ in the wet carbonation chamber. The wet carbonation was conducted at the normal indoor air condition (~25 °C and 1 atm) [8]. One of each nano-adsorbent was placed in the chamber without stirring (non-forced sample, N) and the other was agitated by a magnetic stirrer at 100 rpm (forced sample, F). Pure CO_2_ gas (99.99% purity) was conditioned at ~100%RH at 25 °C by passing through a bubbler filled with D.I. water at 4 lpm using a mass flow controller (Alicat Co., Ltd, Tucson, AZ, USA). Then, the humidified CO_2_ was continuously introduced into the wet carbonation chamber. Farajzadeh et al. reported the characteristic time of dissolving of gas phase of CO_2_ (~99.9%) (CO_2_ + H_2_O → H_2_CO_3_) into water through the CO_2_-water interface at ~100%RH, room temperature (25 °C) was <25 s [28], which is much shorter than our experimental time scale (every 2 h sampling, up to 12 h). In addition, H_2_CO_3_ in the MgO nano-adsorbent slurries could be fully developed during the wet carbonation because the thicknesses of MgO slurries in the petri dishes were negligible compared to the height of carbonation chamber. The evaporation of waters in the slurries was also prohibited by controlling the relative humidity of gas environment in the chamber as ~100%RH through the wet carbonation processes. The intermediate carbonate samples for each MgO nano-adsorbent were taken every 2 h, up to 12 h. After the sampling, 1 mL D.I. water was added to maintain the volume of slurry. All carbonate samples were immediately dried in the oven at 80 °C for 24 h.

### 2.2. Analyses of MgO Nanoparticles and the Carbonate Products

The chemical composition and crystallinity of pristine and carbonate samples (N and F) for each MgO nano-adsorbent were analyzed by X-ray diffraction (XRD, Xpert3 Powder X-ray diffractometer, Malvern Panalytical Instrument, Ltd, Malvern, UK) with Cu Kα radiation from 2θ = 5 to 90°. The morphological changes in the MgO nano-adsorbents during the wet carbonations were investigated by a field emission electron scanning microscopic (FE-SEM, JSM-7900F, JEOL, Ltd, Akishima, Japan). The formation of functional groups on the carbonate samples during the wet carbonation was investigated by Fourier-transform infrared spectroscopy (FT-IR, FT/IR-4100, JASCO, Inc, Hachiojo, Japan) in attenuated total reflection (ATR) mode. The crystallite sizes of the MgO nano-adsorbents were calculated by the Scherrer formula $(\tau =\frac{K\lambda }{\beta \cos\theta }$) [8]. For the calculation of crystallite sizes, the shape factor (K) was selected as 0.9 for all samples. The X-ray wavelength for X-ray diffraction measurement was 1.54 Å. The surface areas, total pore volumes and average pore diameters of the MgO nano-adsorbents were determined by a Brunauer–Emmett–Teller (BET) method (BELSORP-max II, Microtac MRB) and are summarized in Table 1. The surface area of all three nanoparticles were smaller than the previous studies with porous MgO. However, A-MgO showed relatively large value of surface area (13.125 m^2^/g^−1^) among the 3 kinds of MgO nanoparticles.

### 2.3. Calculation of Adsorbed CO_2_ Mass per Unit Mass of Carbonation Samples

The mass of adsorbed CO_2_ per unit mass of magnesium carbonate samples (M_CO2_) was quantitatively analyzed by a gas chromatography–thermal conductivity detector (GC-TCD, 7890A, Agilent, Technology, Santa Clara, CA, USA) after the carbonation processes. To compare the adsorption amounts of samples, four types of magnesium carbonate minerals (artinite, nesquehonite, hydromagnesite, magnesite anhydrous) were also calculated theoretically based on their molar weights and chemical structures (artinite = 0.2238, nesquehonite = 0.3181, hydromagnesite = 0.3764 and magnesite anhydrous = 0.522 mg CO_2_ per unit mass of each mineral). The calibration curve of each MgO nano-adsorbent was obtained for 5 points of MgO and hydromagnesite (Sigma Aldrich Co., Ltd., St. Louis, MO, USA) mixture with different ratios; 0:10, 3:7, 6:4, 8:2, 10:0 (hydromagnesite: MgO, respectively), and one crosscheck point (5:5) was also analyzed to evaluate the calibration curve. Calibration curve and crosscheck value are described in Supplementary Materials (Figure S6). For quantifying M_CO2_, 10 mg of each carbonate sample was placed in a headspace vial and mixed with 4 mL of hydrochloric acid (HCl, 0.1 N) to fully dissolve CO_2_ in gas phase from the carbonate particles. After vibrating the vial by a vortex mixer and keeping it to obtain gas phase CO_2_ for 2 h, 250 μL overhead gas was extracted from the vial using a gastight syringe (Hamilton®, gastight®, 1005TLL 5mL, Reno, NV, USA), and then injected to the GC-TCD inlet. The GC-TCD analysis was repeated three times for each carbonate sample, and the GC-TCD data were averaged. The adsorbed amount of CO_2_ was calculated by interpolating the averaged GC-TCD data for the carbonate samples based on the calibration curve made by reference materials. Then, M_CO2_ was calculated by dividing the mass of CO_2_ by the mass of carbonated samples (10 mg).

## 3. Results and Discussion

### 3.1. XRD Result and Crystallinity of Carbonated Samples

**Figure 1:**
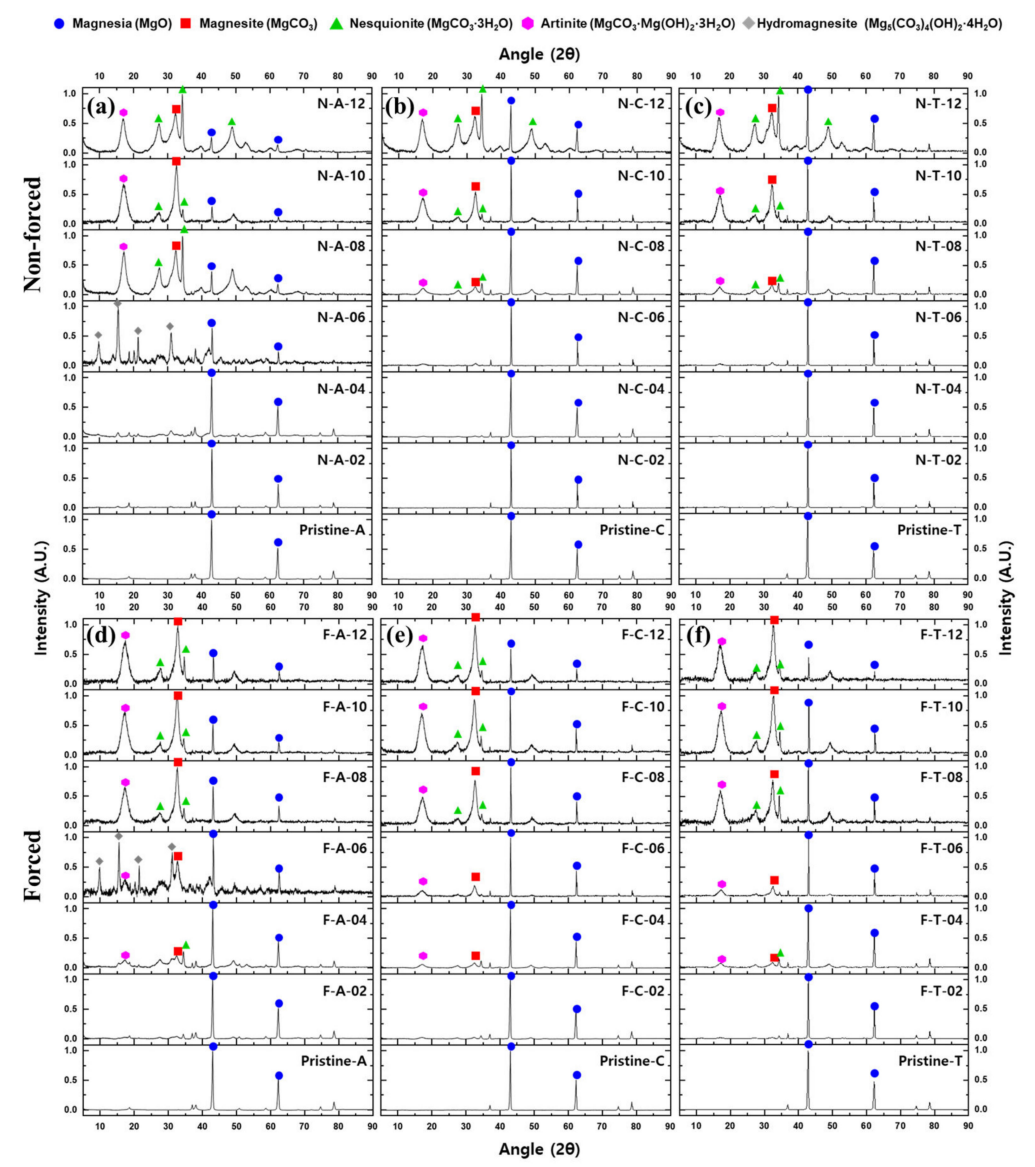
XRD results of the samples for the non-forced (N) wet carbonations of (**a**) A-MgO, (**b**) C-MgO and (**c**) T-MgO as well as forced (F) wet carbonations of (**d**) A-MgO, (**e**) C-MgO and (**f**) T-MgO.

**Figure 2:**
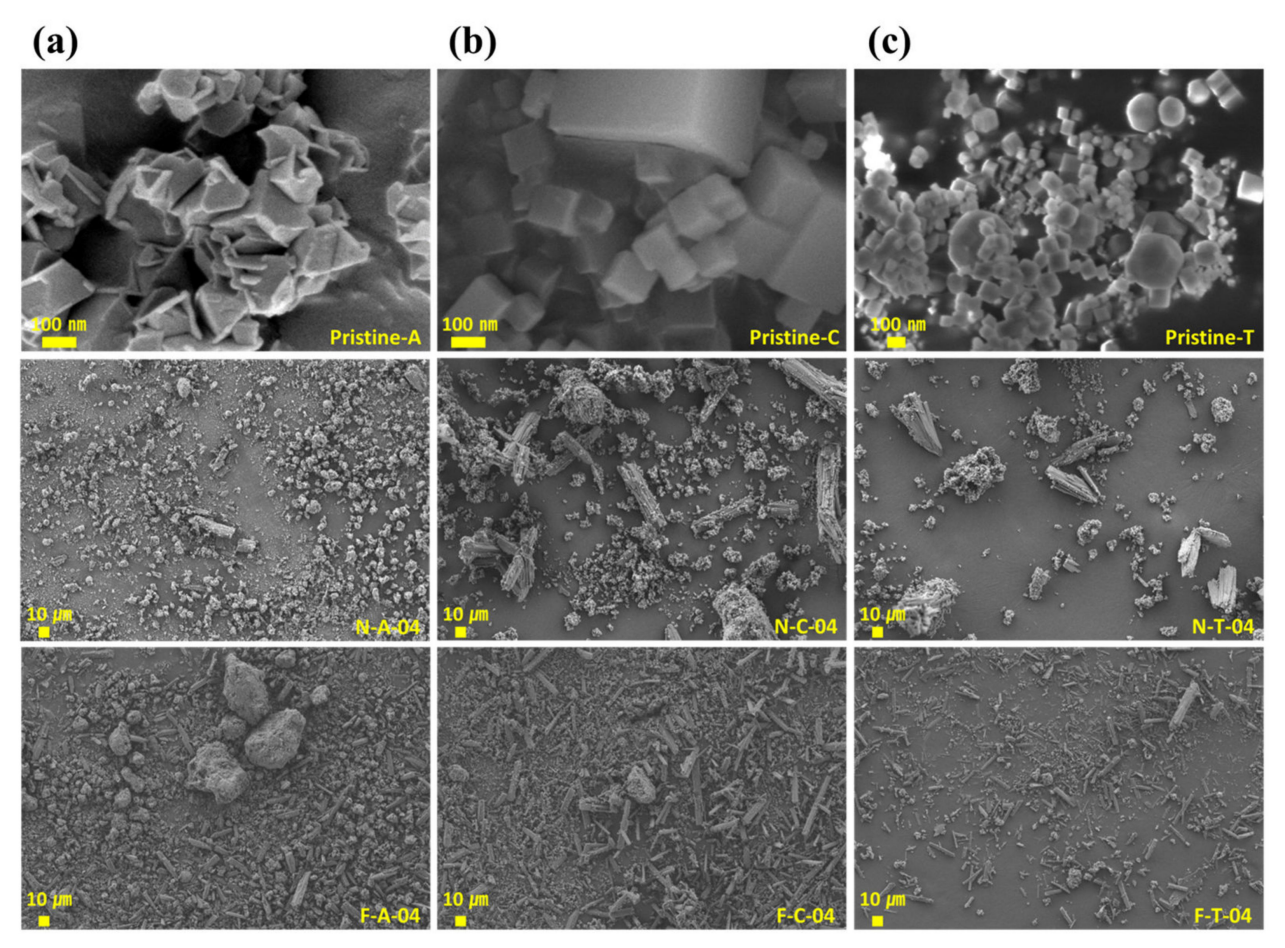
Morphological changes in the 3 MgO nano-adsorbents between pristine and 4 h carbonation products through the forced (F) or non-forced (N) wet carbonations for (**a**) A-MgO, (**b**) C-MgO and (**c**) T-MgO analyzed by FE-SEM (scale bars: 100 nm for pristine particles and 10 μm for carbonated samples) (SEM images at full carbonation time conditions are in the Supplementary Materials) SI).

Figure 1 shows XRD patterns of the N- and F-carbonate samples of the three MgO nano-adsorbents. In the cases of N-carbonation as shown in the upper side of Figure 2, all the MgO nano-adsorbents (A-, C- and T-MgO) started to show the carbonate products after 8 h of wet carbonation. Magnesite (MgCO_3_) and hydrated magnesium carbonates (nesquehonite (MgCO_3_·3H_2_O) and artinite (MgCO_3_·Mg(OH)_2_·3H_2_O)) [29] were formed until 12 h; however, the formation of carbonate products did not finish until after 12 h. Compared to the C- and T-MgO, A-MgO showed hydromagnesite (Mg_5_(CO_3_)_4_(OH)_2_·4H_2_O) in the 6 h carbonate sample as an intermediate product. At the same time, the intensity of the MgO peak at 2θ = 42° for A-MgO reduced quickly after 6 h, while C- and T-MgO showed clear and sharp pristine MgO peaks. Compared to the N-carbonation samples, the formation of nesquehonite, artinite and magnesite was facilitated by the F-carbonation and was observed in the samples after 4 h as shown in the bottom side of Figure 1, regardless of the kind of MgO nano-adsorbents. The F-carbonation processes for the three MgO nano-adsorbents were almost finished after 8 h, which can be seen in the XRD patterns between 8 and 12 h. Interestingly, the MgO peak was reduced for the 12 h F-carbonation samples of C- and T-MgO and relatively lower than the magnesite peak. This phenomenon would be observed if the N-carbonation proceeded further. A-MgO had a different carbonation pathway than other nano-adsorbents; in particular, the hydromagnesite formation with nesquehonite shown in the 6 h N- and F-carbonation samples. Figures S1 and S4 show a mixture of pillar-shaped nesquehonite and flake-shaped hydromagnesite particles [30,31,32]. The number of pillar-shaped nesquehonite particles increased with the carbonation time, as depicted in Figure S4. However, the hydromagnesite particles were not able to be seen very well from the XRD and SEM images for C- and T-MgO.

Table 2 summarizes the crystallite sizes of nesquehonite (2θ = 34°) produced by the carbonation of the MgO nano-adsorbents, which were calculated from the XRD data. The peaks for nesquehonite were not observed in the XRD data at the beginning of the N-carbonation, and the first observation of the peaks depended on the MgO nano-adsorbents. The nesquehonite crystalline sizes of A-MgO, C-MgO and T-MgO increased from 0 to 21.1 nm, 0 to 20 nm and 0 to 18.6 nm, over 8 h, respectively. In the case of forced wet carbonation, the crystalline growth of the nesquehonite began after 4 h for all three kinds of MgO absorbents, the size of which increased from 0 to 17.6~18.6 nm. However, the crystalline growth started after 8 h in the non-forced wet carbonation. Such a starting point of growth means the forced wet carbonation promoted the CO_2_ absorbing reaction of all MgO nanoparticles.

### 3.2. Morphological Changes in MgO Nano-Adsorbents

Figure 2 shows the morphological changes from pristine particles to the carbonate products after 4 h of the N- and F-carbonation processes. Pristine A-, C- and T-MgO nano-adsorbents originally had octahedral, cubic and spherical shapes, respectively. After the 4 h wet carbonation, the carbonate products showed significant differences between the N- and F-carbonation processes, which implies that the processes were accelerated by the F-carbonation. F-carbonation samples showed more pillar-shaped nesquehonite [30,32,33], while N-carbonation ones showed more agglomerates of primary particles. This means that F-carbonation accelerated carbonate formation, which is also shown in Figure 1. The F-carbonation processes also produced some agglomerate particles (several tenth~hundredth μm) for the MgO nano-adsorbents, which were made by the collision of primary particles due to the stirring. For N-carbonation, shape changes in the carbonate products caused by incomplete carbonation were observed until the 12 h samples. However, the F-carbonation samples showed almost completed carbonate particles after 8 h samples for all three of the MgO nano-adsorbents (Figures S1–S3). These results also support the findings in the XRD results in Figure 2 and the crystallite size changes in Table 2.

### 3.3. FT-IR Result with Different Functional Groups

**Figure 3:**
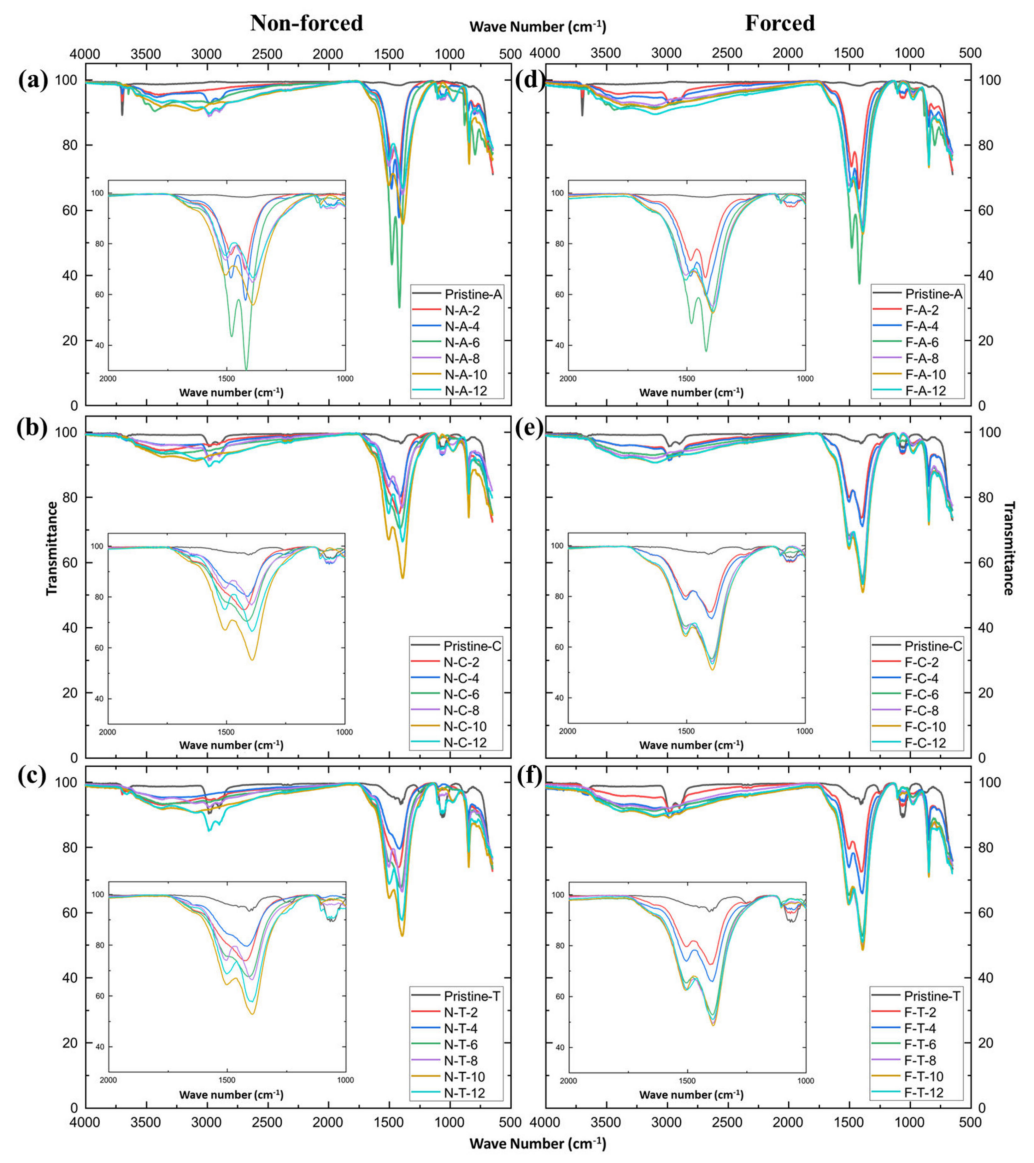
FT-IR spectra results of the pristine MgO nanoparticles and the products after non-forced wet carbonations of (**a**) A-MgO, (**b**) C-MgO and (**c**) T-MgO as well as forced wet carbonations of (**d**) A-MgO, (**e**) C-MgO and (**f**) T-MgO.

Figure 3 shows FT-IR spectra of the three MgO nano-adsorbents during the N- and F-carbonation processes. The inserts in each FT-IR spectrum between 1000 and 2000 cm^−1^ were plotted together to investigate the formation of bicarbonate (HCO_3_^−^) during both N- and F-carbonation. First, the pristine A-MgO nano-adsorbent showed a hydroxyl (OH^−^) peak at 3700 cm^−1^, which was not found in the FT-IR spectra for C- and T-MgO. The hydroxyl peak disappeared during the wet carbonation processes. This means that the A-MgO nano-adsorbent partially contained Mg(OH)_2_ and was transformed into carbonates during the wet carbonation [8]. However, C- and T-MgO synthesized through the aerosol methods in this study maintained their original MgO statuses well.

On the other hand, C- and T-MgO showed similar FT-IR results to the XRD results in Figure 1. During the of wet carbonation, two main peaks at 1390 cm^−1^ (CO_3_^2−^ group) and 1505 cm^−1^ (MgCO_3_ from unidentate) increased with the carbonation time [34]. However, different carbonation times were needed to fully develop these two peaks depending on the carbonation processes (N- vs. F-carbonation). While F-carbonation showed these peaks after 2 h of carbonation and only the transmittance was changed, 6 h of N-carbonation time was needed for these two main peaks to appear in the carbonate products of C- and T-MgO nano-adsorbents.

Once the wet carbonation was started, hydrated magnesium carbonates were produced, showing H_2_O and CO_3_^2−^ bands in the FT-IR spectra, as discussed on the XRD data. For instance, the intensity of the CO_3_^2−^ band at 845 cm^−1^ [35,36,37] was increased for all three of the MgO nano-adsorbents as the wet carbonation was conducted. In addition, the A-MgO nano-adsorbent showed the shape change in the 800–880 cm^−1^ band until 6 h of carbonation, which matched well with the change in the XRD patterns for A-MgO. As shown in the inserts in Figure 3a,b, the FT-IR spectrum bands at 1420 and 1480 cm^−1^ between 2 and 6 h of N- and F- carbonation were increased and approached the highest intensity after 6 h of carbonation. The bands were reported to indicate both CO_3_^2−^ and HCO_3_^−^ on the hydromagnesite surfaces [35]. However, the bands were broadened to 1395 and 1505 cm^−1^ for the 8 h to 12 h carbonation samples of both N- and F-carbonation. Furthermore, N-carbonation showed different results in that the transmittances of the main peaks (1390 and 1505 cm^−1^) decreased at 12 h carbonation. While the F-carbonation was saturated after 8 h of carbonation, N-carbonation kept increasing the transmittance until 10 h, and the peak dropped at 12 h.

When comparing the peaks at 800–880 cm^−1^ in Figure S5, the forced carbonation processes of all three MgO nano-adsorbents were saturated at around 6 h. The left side of Figure S5 shows with details that the carbonated peak in 845 cm^−1^ overlapped from 6 h to 12 h, while the H_2_O peaks at around 1650 cm^−1^ [35,38,39] on the right side of Figure S5 also overlapped at the same time.

### 3.4. Quantitative Comparison of M_CO2_

**Figure 4:**
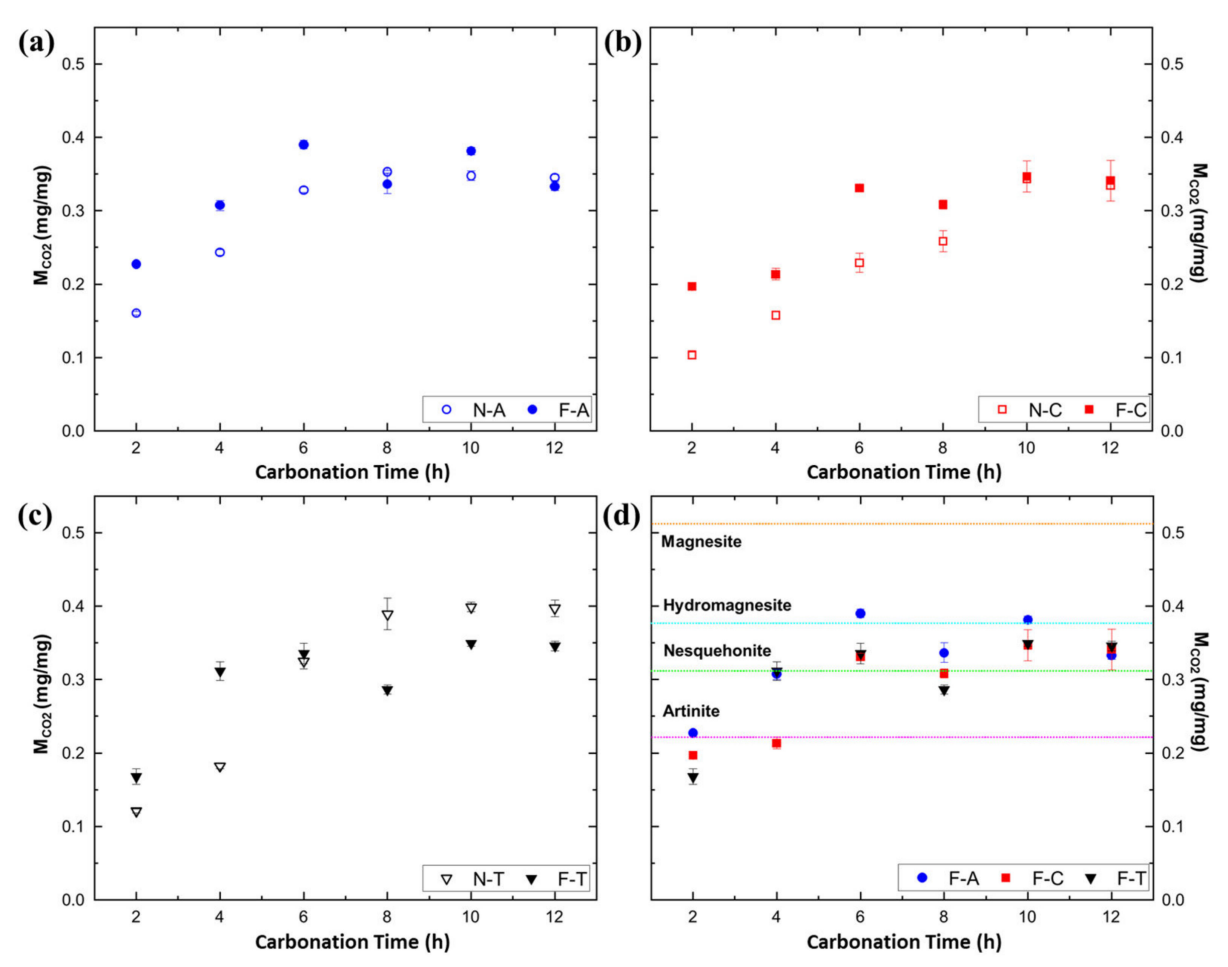
Adsorbed CO_2_ masses per unit mass (M_CO2_) of the forced (F) or non-forced (N) wet carbonation products analyzed by GC-TCD for (**a**) A-MgO, (**b**) C-MgO and (**c**) T-MgO. (**d**) M_CO2_ of the F-carbonation products of the 3 MgO nano-adsorbents were compared with theoretical maximum CO_2_ adsorption masses of the possible MgO carbonates such as magnesite, hydromagnesite, nesquehonite and artinite.

Figure 4 shows the amounts of adsorbed CO_2_ (M_CO2_) in the unit mass of carbonated samples analyzed by GC-TCD. The differences in the M_CO2_ between F- and N-carbonation samples are also summarized in Table 3 according to the carbonation time. Overall, the F-carbonation samples showed larger amounts of M_CO2_ in the sample compared to the N-carbonation ones in the early stage of the carbonation processes. F-carbonation increased the magnitude of M_CO2_ for all three of the MgO nano-adsorbents until the 6 h samples, compared to N-carbonation samples, as shown in Figure 4a–c. The F-carbonation of the three MgO nano-adsorbents showed almost similar magnitudes of M_CO2_ from 6 h, as also described in FT-IR, and the averages and standard deviations of M_CO2_ of F-carbonation between 6, 8, 10 and 12 h were as follows: A-MgO: 0.360 ± 0.026, C-MgO: 0.331 ± 0.015 and T-MgO: 0.329 ± 0.025. However, N-carbonation needed relatively more carbonation time to show these flat saturations and the three MgO nano-adsorbents showed different times for this saturation. As also mentioned in a previous study [8], C-MgO needed the longest carbonation time of 10 h, while A-MgO required a time almost similar with F-carbonation (6 h). The M_CO2_ for the three F-carbonation samples with the theoretical maximum amounts of M_CO2_ by different kinds of hydrated magnesium carbonates (artinite = 0.2238, nesquehonite = 0.3181, hydromagnesite = 0.3764 and magnesite anhydrous = 0.522 mg-CO_2_/mg of each standard carbonate) are described in Figure 4d. The M_CO2_ amounts of the three MgO nano-adsorbents placed between hydromagnesite and nesquehonite after 6 h of wet carbonation, and the F-carbonation processes were finished as shown by the FT-IR and XRD data. This implies that most of the nano-adsorbent particles were transformed into hydrated magnesium carbonates, rather than anhydrous MgCO_3_, due to the reaction temperature and moderate pressure of CO_2_ gases [33].

After 8 h, the M_CO2_ amounts of the N-carbonation samples were closer to the values of F-carbonation for the A- and C-MgO nano-adsorbents. However, T-MgO showed higher CO_2_ adsorption for the N-carbonation samples than the F-carbonation ones. The T-MgO nano-adsorbent showed an average M_CO2_ value of 0.395 ± 0.004 between 8~12 h of N-carbonation. This magnitude was much larger than the F-carbonation of T-MgO between 6~12 h (0.344 ± 0.006) even after eliminating the odd value (8 h). One of the possible reasons for this distinct characteristic is that T-MgO nanoparticles have more structural defects than C-MgO nanoparticles as previous studies mentioned [24,25,27]. From the previous research, the defects of the MgO nanoparticles were shown to be the active basic sites that reacted with acidic CO2 gas and had lower adsorption and regeneration temperatures [14,40,41]. At the same time, similar to previous studies [30,31,32], SEM of the N-carbonation of the T-MgO nano-adsorbent only showed hydromagnesite, which has a larger M_CO2_ value than nesquehonite (Figure S3). Furthermore, in Figure 1, the XRD peaks of the F-carbonation of T-MgO showed less nesquehonite than for N-carbonation. This could be evidence that even though F-carbonation could accelerate the mineral carbonation, F-carbonation also became a barrier for the crystallite growth of hydromagnesite and other more carbonate-containing products from artinite to nesquehonite and hydromagnesite [34].

## 4. Conclusions

In this study, three kinds of MgO nano-adsorbents synthesized by aerosol methods were investigated to figure out the effects of the external force on the carbonation performance at room temperature by comparing the characteristics of forced (F) and non-forced (N) carbonation samples. Due to the external force, F-carbonation processes showed carbonation products (artinite or nesquehonite) 4 h earlier than N-carbonation ones for all MgO nano-adsorbents. In addition, F-carbonation processes for the MgO nano-adsorbents were finished after 8 h, although N-carbonation processes were not finished until after 12 h. Even though the pristine morphologies of the MgO nano-adsorbents were different depending on the synthesis methods, they had similar wet carbonation capacities based on the GC-TCD analysis. The GC-TCD results for the MgO nano-adsorbents imply that the MgO nanoparticles were transformed into hydrated magnesium carbonates, such as hydromagnesite and nesquehonite, because F- and N-carbonation were conducted as wet carbonation processes. In other words, the F-carbonation after stirring the MgO adsorbent slurry has faster adsorption kinetics compared to N-carbonation. However, F-carbonation limited the crystalline growth of hydrated magnesium carbonates effectively as shown by the GC-TCD and XRD results described above.

This wet carbonation technology could be applied to capture CO_2_ in the flue gas, atmosphere and indoor air as a direct air capture technology. Applying the external force on the wet carbonation can enhance the CO_2_ capture efficiency of the direct air capture using slurry-type MgO nano-adsorbents. In addition, the aerosol-processed MgO nano-adsorbents do not produce wastewater for post-treatments and harmful gaseous pollutants during the synthesis processes. The lower regeneration temperature of MgO compared with CaO can also save energy and costs as well as extend the lifetime of the adsorbents.

For direct air capture applications, the carbon capture characteristics at CO_2_ concentrations lower than the test gas in this study (for example, <1000 ppm CO_2_ in the indoor air) need to be investigated further. Moreover, the dry carbonation characteristics of the aerosol-processed MgO nano-adsorbents at elevated temperatures can increase the CO_2_ capture efficiency and prevent fungi formation in the indoor air caused by the water used in the wet carbonation during the direct air capture in the indoor air environment. This study on the dry carbonation characteristics also makes it easier to understand the mechanism of the carbonation process. Understanding the carbonation mechanism can be fundamental for manipulating the MgO-based CO_2_ adsorbents depending on their future applications.

## Figures and Tables

**Table 1:** Physical characteristics of three pristine MgO nano-adsorbents measured by BET method (Four significant digits).

	A-MgO (Commercial Nanopowder)	C-MgO (Self-Combustion)	T-MgO (Flame Metal Combustion)
Surface Area (m^2^/g^−1^)	13.13	10.94	4.059
Total Pore Volume (cm^3^/g^−1^)	0.1119	0.02878	0.02716
Average Pore Diameter (nm)	34.10	10.52	26.76

**Table 2:** Crystallite sizes of nesquehonite of three MgO nano-adsorbents at different carbonation times (unit: nm).

(Unit: nm)	A-MgO (Commercial Nanopowder)		C-MgO (Self-Combustion)		T-MgO (Flame Metal Combustion)	
Carbonation Hour (h)	N	F	N	F	N	F
0 (Pristine)	0	0	0	0	0	0
2	0	0	0	0	0	0
4	0	17.6	0	18.6	0	17.6
6	0	10.0	0	-	0	17.6
8	21.1	14.5	20.0	13.9	18.6	18.6
10	7.2	12.7	21.3	20.0	15.1	16.7
12	16.7	21.3	16.8	17.8	17.6	16.7

**Table 3:** Adsorbed CO_2_ mass per unit mass of carbonated MgO samples (M_CO2_) (mg/mg) through GC-TCD analysis and the differences of M_CO2_ (ΔM_CO2_) between non-forced (N) and forced (F) wet carbonation samples.

(Unit: mg/mg)	A-MgO (Commercial Nanopowder)		C-MgO (Self-Combustion)		T-MgO (Flame Metal Combustion)	
Carbonation Hour (h)	N	F	N	F	N	F
2	0.161 ± 0.002	0.227 ± 0.002	0.102 ± 0.006	0.195 ± 0.006	0.121 ± 0.003	0.168 ± 0.010
	ΔM_CO2_ = 0.067		ΔM_CO2_ = 0.093		ΔM_CO2_ = 0.047	
4	0.243 ± 0.004	0.307 ± 0.007	0.156 ± 0.004	0.212 ± 0.008	0.182 ± 0.003	0.312 ± 0.013
	ΔM_CO2_ = 0.064		ΔM_CO2_ = 0.056		ΔM_CO2_ = 0.129	
6	0.328 ± 0.004	0.390 ± 0.006	0.227 ± 0.013	0.329 ± 0.005	0.325 ± 0.021	0.287 ± 0.006
	ΔM_CO2_ = 0.062		ΔM_CO2_ = 0.102		ΔM_CO2_ = 0.011	
8	0.353 ± 0.002	0.337 ± 0.013	0.257 ± 0.014	0.307 ± 0.006	0.389 ± 0.021	0.287 ± 0.006
	ΔM_CO2_ = −0.016		ΔM_CO2_ = 0.050		ΔM_CO2_ = −0.103	
10	0.348 ± 0.006	0.381 ± 0.005	0.342 ± 0.004	0.345 ± 0.021	0.399 ± 0.006	0.349 ± 0.004
	ΔM_CO2_ = 0.034		ΔM_CO2_ = 0.003		ΔM_CO2_ = −0.050	
12	0.345 ± 0.004	0.332 ± 0.005	0.333 ± 0.005	0.339 ± 0.027	0.397 ± 0.011	0.346 ± 0.006
	ΔM_CO2_ = −0.012		ΔM_CO2_ = 0.006		ΔM_CO2_ = −0.051	

## Data Availability

Not applicable.

## Supplementary Materials

The following supporting information can be downloaded at: https://www.mdpi.com/article/10.3390/nano13020281/s1, Figure S1: Morphological changes in the A-MgO nano-adsorbents during the forced (F) or non-forced (N) wet carbonation at different carbonation times (scale bars: 100 nm, 200 nm and 10 μm depending on the images); Figure S2: Morphological changes in the C-MgO nano-adsorbents during the forced (F) or non-forced (N) wet carbonation at different carbonation times (scale bars: 100 nm, 200 nm and 10 μm depending on the images); Figure S3: Morphological changes in the T-MgO nano-adsorbents during the forced (F) or non-forced (N) wet carbonation at different carbonation times (scale bars: 100 nm, 200 nm and 10 μm depending on the images); Figure S4: Morphological changes in the A-MgO nanoparticles during the non-forced wet carbonation. (scale bars: 100 μm from larger image and 10 (N-A-6) and 1 μm (N-A-4 and N-A-8) from smaller image); Figure S5: Detailed FT-IR spectra for the 3 forced carbonation samples in the ranges of 800–1000 cm^−1^ (left, HCO_3_^−^) and 1550–1750 cm^−1^ (right, H_2_O); Figure S6: Calibration curve of each MgO nano-adsorbent with different hydromagnesite ratio in 10 mg powder and crosscheck value, which was 5 mg of hydromagnesite.
