# Supplementary material for: Forced Mineral Carbonation of MgO Nanoparticles Synthesized by Aerosol Methods at Room Temperature
Source: Nanomaterials (Basel). 2023 Jan 9;13(2):281. doi: 10.3390/nano13020281 (PMC9862497; doi:10.3390/nano13020281)
Supplement: Supplementary file 1 [file nanomaterials-13-00281-s001.zip › nanomaterials-2151751-supplementary.pdf]

Supplementary Materials

Forced Mineral Carbonation of MgO Nanoparticles Synthesized by Aerosol Methods at Room Temperature

Kyungil Cho [1], Yeryeong Kang [1], Sukbyung Chae [2,*] and Changhyuk Kim [1,*]

[1] School of Civil and Environmental Engineering, Pusan National University, Busan 46241, Republic of Korea
[2] Department of Mechanical Engineering, Korea University of Technology and Education, Cheonan 31253, Republic of Korea
* Correspondence: schae@koreatech.ac.kr (S.C.); changhyuk.kim@pusan.ac.kr (C.K.)

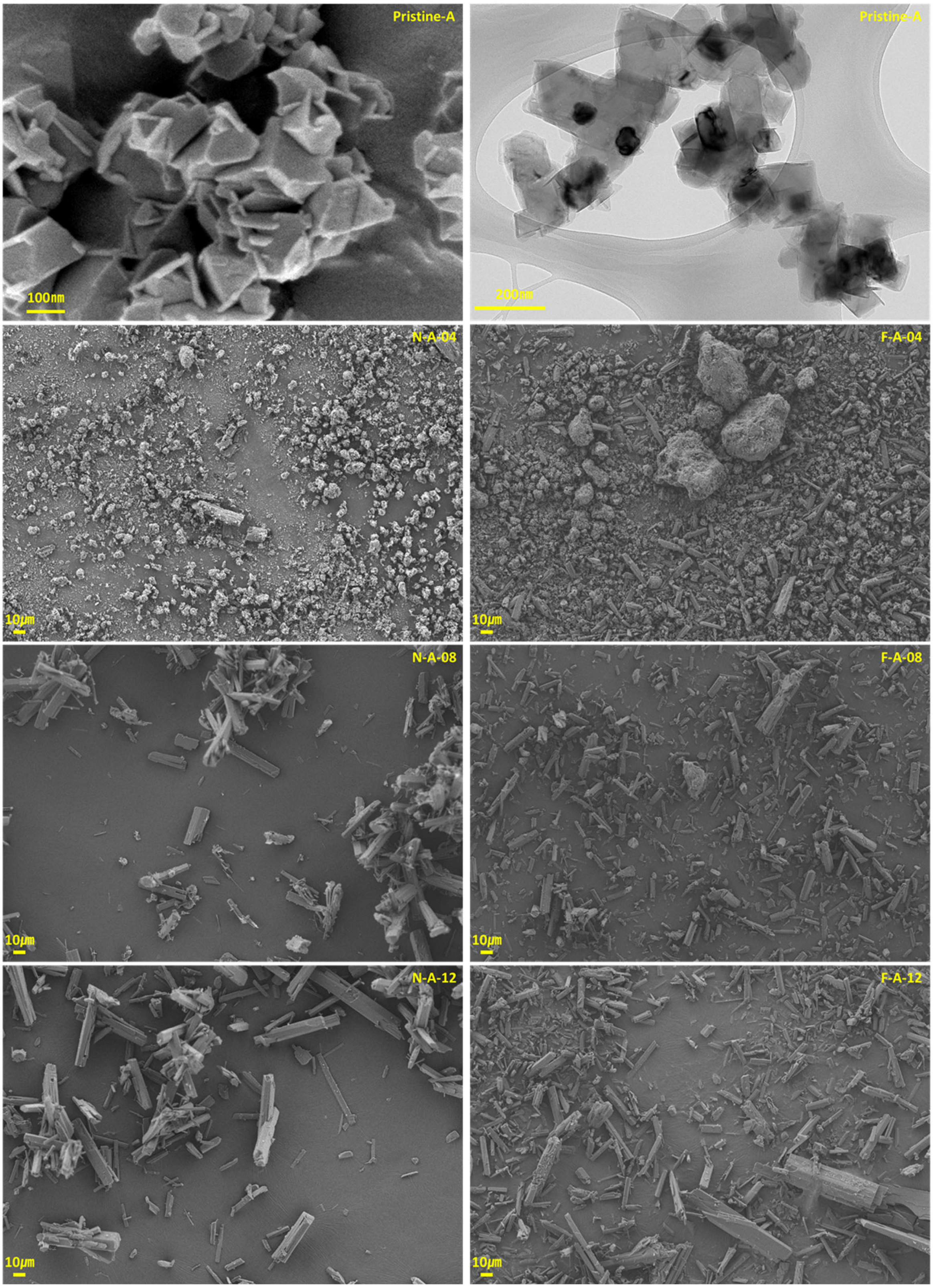

Figure S1. Morphological changes for the A-MgO nano-adsorbent during the forced (F) or non-forced (N) wet carbonation at different carbonation times (scale bars: 100 nm, 200 nm, and 10 μm depending on the images)

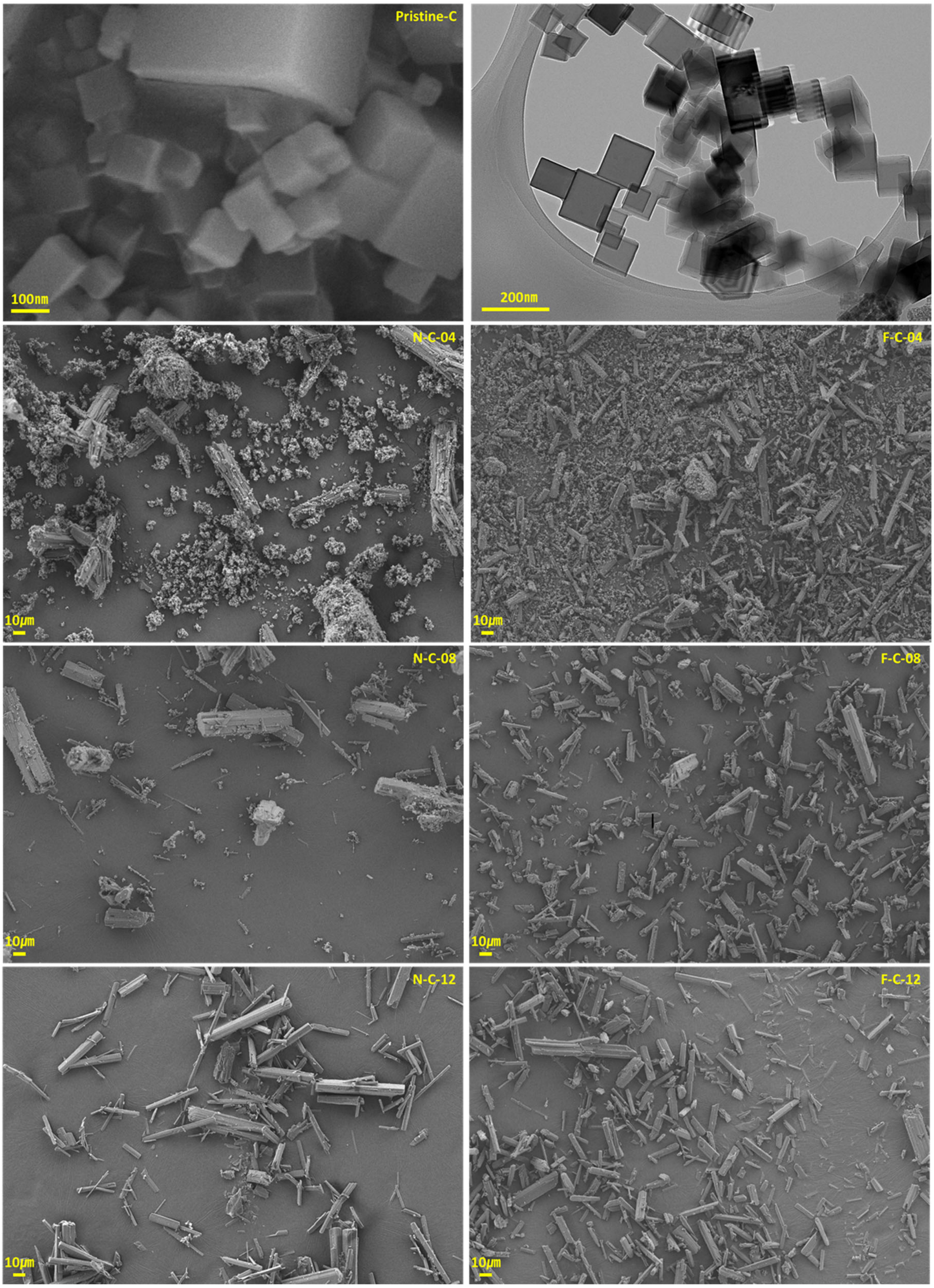

Figure S2. Morphological changes for the A-MgO nano-adsorbent during the forced (F) or non-forced (N) wet carbonation at different carbonation times (scale bars: 100 nm, 200 nm, and 10 μm depending on the images)

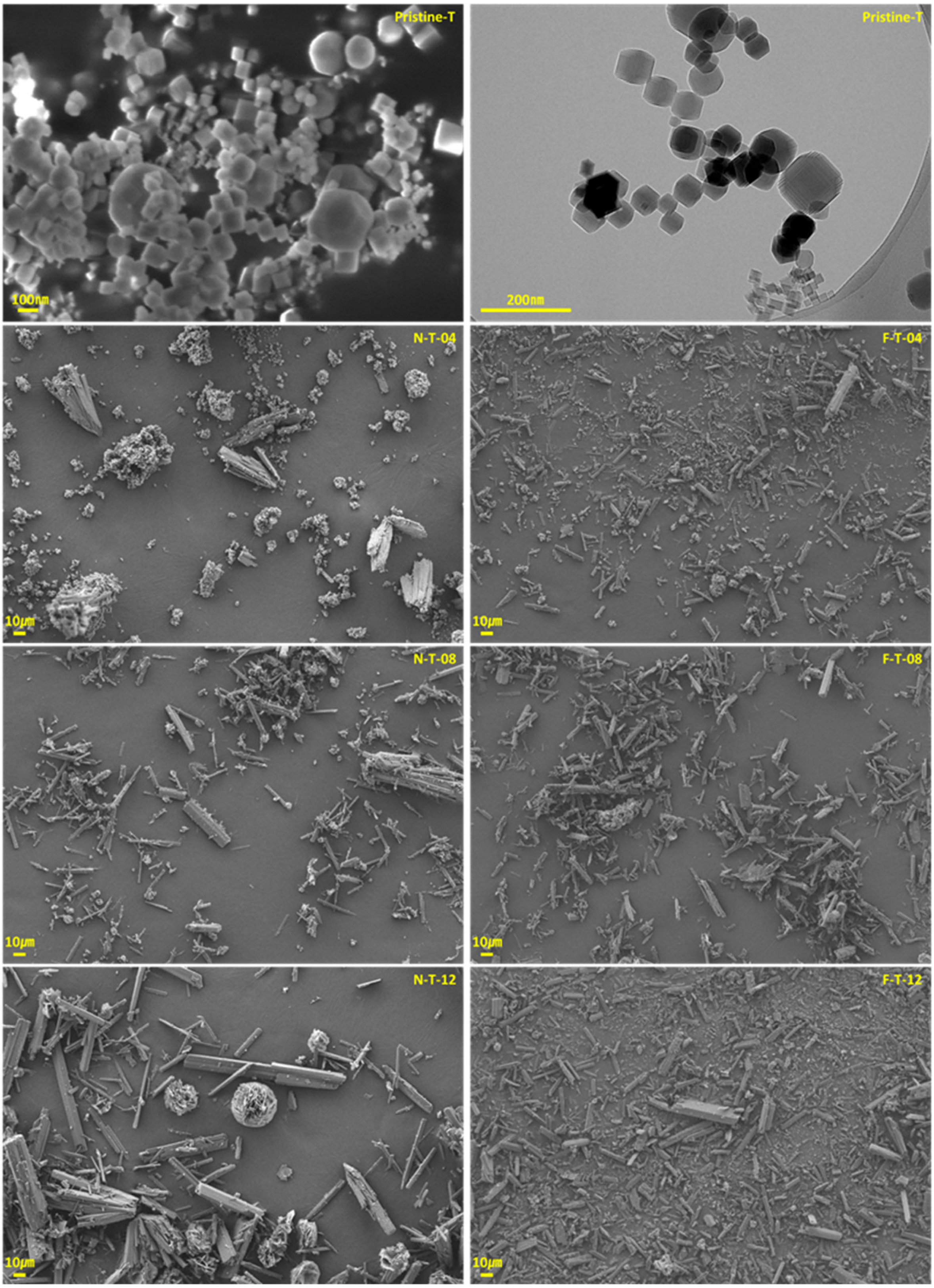

Figure S3. Morphological changes for the A-MgO nano-adsorbent during the forced (F) or non-forced (N) wet carbonation at different carbonation times (scale bars: 100 nm, 200 nm, and 10 μm depending on the images)

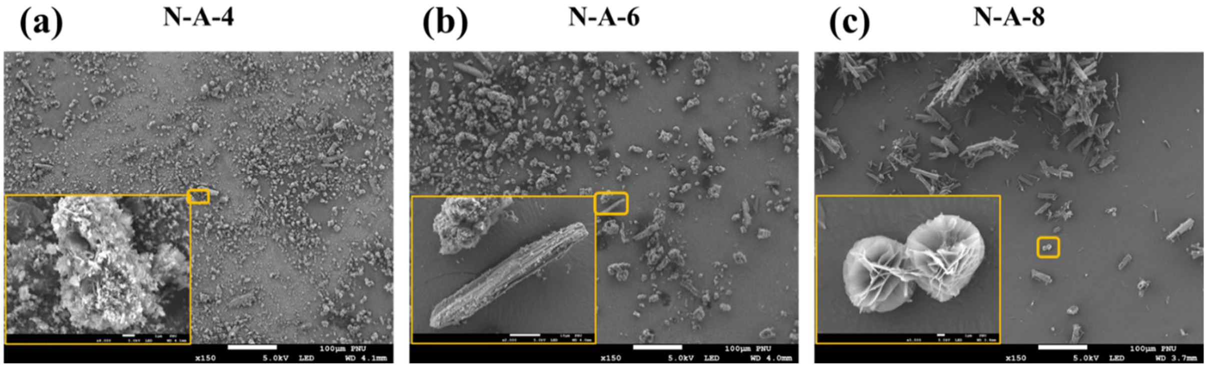

Figure S4. Morphological changes of the A-MgO nanoparticles during the Non-forced wet carbonation. (scale bars: 100 ㎛ for all three larger image (N-A-4, 6, 8) and 10 (N-A-6) and 1 ㎛ (N-A-4 and N-A-8) for Smaller image)

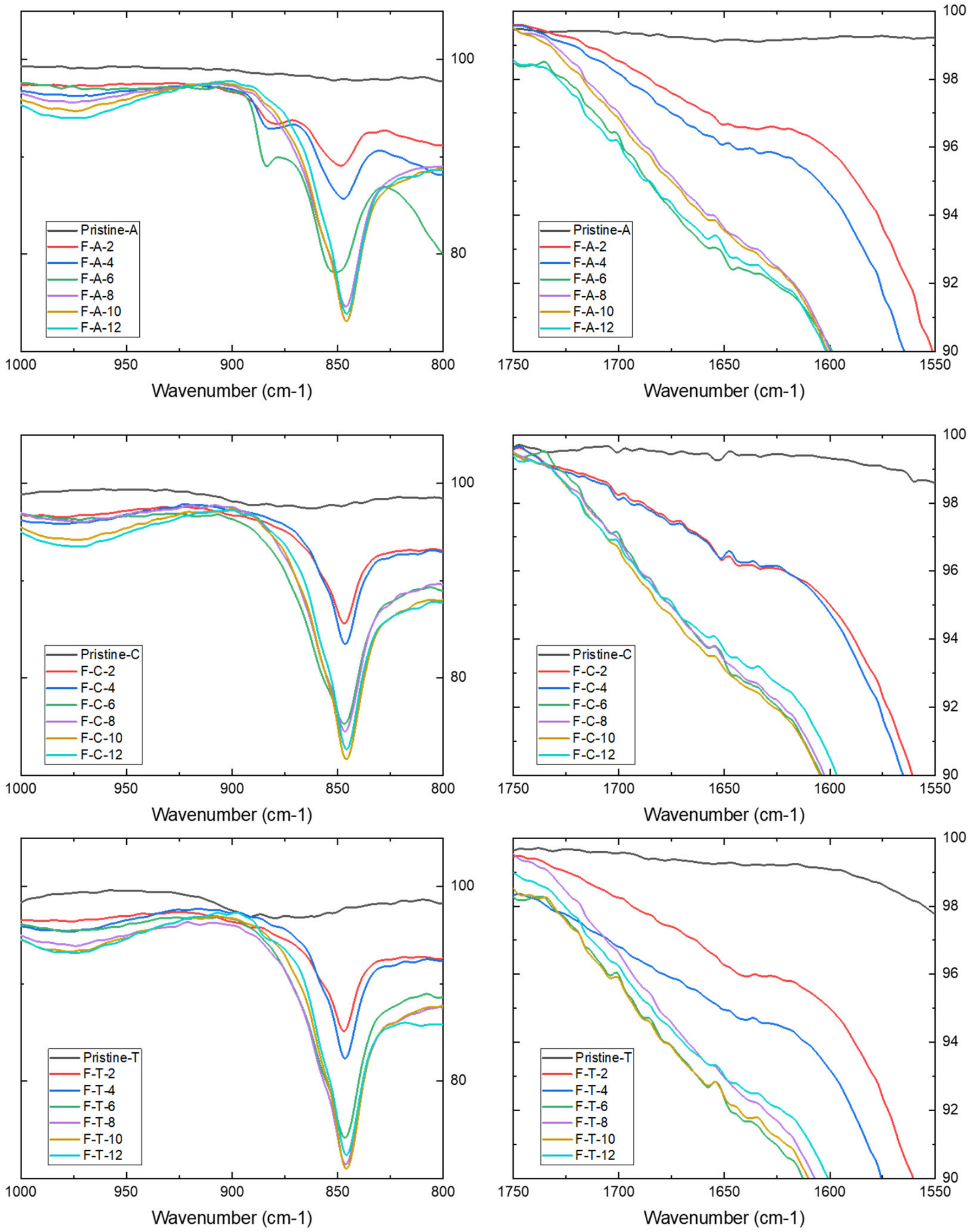

Figure S5. Detailed FT-IR spectra for the 3 forced carbonation samples in the ranges of 800 – 1000 cm^{-1} (left, HCO_3^-) and 1550 – 1750 cm^{-1} (right, H_2O)

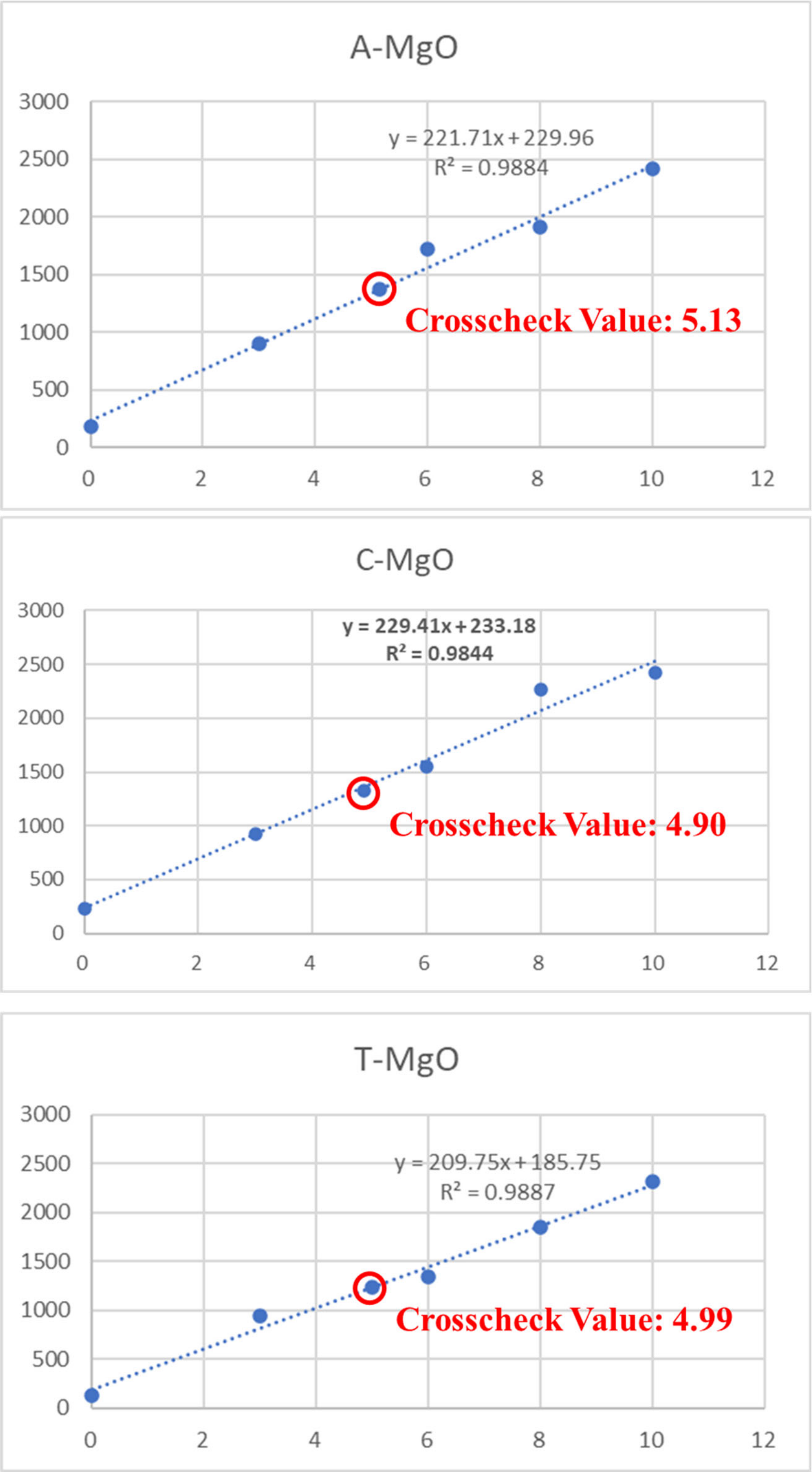

Figure S6. Calibration curve of each MgO nano-adsorbent with different Hydromagnesite ratio in 10mg powder and crosscheck value which was 5 mg of Hydromagnesite
